# A new species of stygobitic snail in the genus *Antrorbis* Hershler & Thompson, 1990 (Gastropoda, Cochliopidae) from the Appalachian Valley and Ridge of eastern Tennessee, USA

**DOI:** 10.3897/zookeys.898.46917

**Published:** 2019-12-10

**Authors:** Nicholas S. Gladstone, Kathryn E. Perez, Evelyn B. Pieper, Evin T. Carter, Katherine E. Dooley, Nathaniel F. Shoobs, Annette S. Engel, Matthew L. Niemiller

**Affiliations:** 1 Department of Zoology, Southern Illinois University, Carbondale, IL 62901, USA; 2 Department of Biology, The University of Texas Rio Grande Valley, Edinburg, TX, USA; 3 Department of Ecology and Evolutionary Biology, University of Tennessee, Knoxville, Knoxville, TN 37996, USA; 4 Environmental Sciences Division, Oak Ridge National Laboratory, Oak Ridge, TN 37830, USA; 5 Department of Biological Sciences, The University of Alabama in Huntsville, Huntsville, AL 35899, USA; 6 Department of Biodiversity, Earth and Environmental Science, Drexel University, Philadelphia, PA 19104, USA; 7 Department of Malacology, Academy of Natural Sciences of Drexel University, Philadelphia, PA 19104, USA; 8 Department of Earth and Planetary Sciences, University of Tennessee, Knoxville, TN 37996, USA

**Keywords:** *
Antrorbis
*, cavesnail, Cochliopidae, stygofauna, systematics

## Abstract

A new species of cave snail (Littorinimorpha: Cochliopidae) in the genus *Antrorbis* is described from the dark zone of two caves in the Appalachian Valley and Ridge province in eastern Tennessee, United States. The Tennessee Cavesnail, *Antrorbis tennesseensis* Perez, Shoobs, Gladstone, & Niemiller, **sp. nov.** is distinguished from its only known congener, *Antrorbis breweri*, by the absence of raised tubercles on its finely spirally striate protoconch, and its unique radular formula. Moreover, *A. tennesseensis* is genetically distinct from *A. breweri* based on substantial divergence at the mitochondrial CO1 locus. This is the first cavesnail to be described from the Appalachian Valley and Ridge (AVR) physiographic province in the state of Tennessee, which previously represented a substantial gap in the distribution of stygobitic (i.e., aquatic, subterranean-obligate) gastropods.

## Introduction

Among the hydrobioid snails (i.e., Hydrobiidae s.l.; Davis 1979) are a morphologically diverse group of minute gastropods living in freshwater subterranean habitats in karstic regions of North America (Hershler and Holsinger 1990; Niemiller et al. 2019). These stygobitic (i.e., aquatic, subterranean-obligate) gastropods are characterized by reduced shell thickness, soft body depigmentation, nearly complete eye reduction, and miniaturization (Hershler and Holsinger 1990; Grego et al. 2019; Prié 2019).

Of the North American stygobitic snails, the Lithoglyphidae initially contained the highest species diversity and has been traditionally divided into several groups on the basis of morphology and geographic distribution: 1) *Phreatodrobia*, *Phreatoceras*, *Balconorbis*, *Stygopyrgus*, and *Texapyrgus*, endemic to the Edwards-Trinity Aquifer System in south and central Texas; 2) *Antrorbis* and *Holsingeria*, found in the Appalachians karst region of the eastern United States; and 3) *Pterides* from northeastern Mexico (Hershler and Longley 1986; Hershler 1989; Hershler and Holsinger 1990; Hershler and Thompson 1990). However, the classification of related groups of minute snails has been recently revised (Wilke et al. 2013). Several genera of stygobitic snails from North America that were members of Lithoglyphidae (*Phreatodrobia*, *Phreatoceras*, *Balconorbis*, *Stygopyrgus*, and *Texapyrgus*) were reassigned to Cochliopidae (Clark 2019a). *Antrorbis*, *Holsingeria*, and *Pterides* were retained in Lithoglyphidae (Clark 2019b), despite no new evidence for these designations. More recent studies of both stygobitic and spring-dwelling gastropods emphasize the need to incorporate molecular evidence in taxonomy and classification due to morphological convergence among snails adapted to these habitats (Delicado 2018; Delicado et al. 2019).

The Appalachians karst region occurs within the Appalachian Valley and Ridge (AVR) physiographic province that extends from southeastern New York to eastern Tennessee, northwestern Georgia, and northeastern Alabama in the eastern United States. The province is situated between the Blue Ridge Mountains to the east and the Appalachian Plateau (specifically the Cumberland Plateau) to the west. Karst terrain in the AVR has developed in valleys of folded and faulted shale and carbonate rocks between parallel ridges of sandstone strata. The Appalachians karst region harbors the highest stygobitic diversity in North America (Niemiller et al. 2019), including five species of hydrobioid snails: three species in the genus *Fontigens* found throughout the Greenbrier Valley of West Virginia and into southwestern Virginia (Holsinger et al. 1976; Holsinger and Culver 1988), one species in the genus *Holsingeria* found in several caves within the Clinch-Powell watershed in Lee County, Virginia, and one species in the genus *Antrorbis* endemic to a single cave system within the Coosa River watershed in DeKalb County, Alabama. No stygobitic snails have been described from the Appalachians karst region of eastern Tennessee to date; however, this region has not received the attention of cave biologists relative to other areas in the state (Niemiller and Zigler 2013) and within the Appalachians karst region of other states (e.g., Culver et al. 2003).

During ongoing cave biological inventory efforts to address the previously identified sampling gaps in the AVR of eastern Tennessee, we discovered three distinct populations of an undescribed stygobitic snail within the Tennessee River watershed of Roane and Knox counties, Tennessee. These snails resembled the Manitou Cavesnail, *Antrorbis breweri*Hershler and Thompson 1990 described from Fort Payne, Alabama, and would represent a ca. 250 km extension in the distribution of the genus (Hershler and Thompson 1990) and a second species in this monotypic genus. After assessment of shell morphology, reproductive anatomy, and molecular barcoding analyses, we describe two of these new populations (Cave Creek Cave and Eblen Cave) as the first stygobitic snail in eastern Tennessee, *Antrorbis tennesseensis* sp. nov. In addition, we tentatively classify a third population (Pedigo Cave) as Antrorbis cf. tennesseensis, reflecting the uncertainty in phylogenetic analyses despite similar morphology. Lastly, we generate a phylogeny to test the position of *Antrorbis* among other stygobitic and non-stygobitic Cochliopidae.

## Materials and methods

### Survey protocol and site descriptions

**Figure 1.**
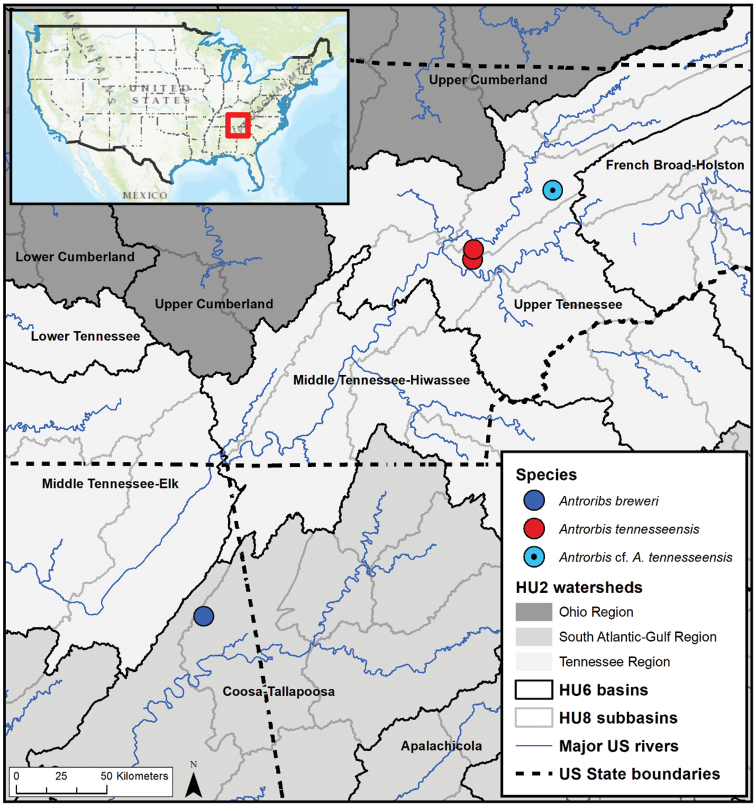
Distribution of *Antrorbis* spp. in relation to USGS hydrologic units: regions (HU2, shaded areas), basins (HU6, solid black lines), and subbasins (HU8, solid grey lines; unlabeled in map). *Antrorbis breweri* occurs within the Upper Coosa subbasin of the South Atlantic-Gulf region, whereas *A. tennesseensis* and Antrorbis cf. tennesseensis occurs in the Watts Bar Lake and Lower Clinch subbasins within the Upper Tennessee basin (Tennessee region), respectively.

Since 2012, the authors have conducted more than 200 biological inventories in caves throughout the AVR in Tennessee and neighboring states (Engel et al. 2016; Niemiller et al. 2016a, b, 2017; Gladstone et al. 2018; Zigler et al. in press) to address previously identified sampling gaps (Niemiller and Zigler 2013). Biological inventories involve systematic visual encounter surveys (VES) for cave life by traversing the cave from entrance to the farthest extent of the explorable passage. Search effort includes lifting rocks and other cover, as well as searching through cobbles, detritus, and organic matter. Typically, each VES consists of 2–7 surveyors, with search effort ranging from 2–5 person-hours per cave visit and depending on the length and extent of the system. General water physiochemistry was assessed in each cave at the time of snail observation and collection using standard, handheld electrode methods for pH and conductivity (Fisher Scientific Accumet AP115 and AP75, respectively), each with temperature. Cave descriptions and location details are maintained by the Tennessee Cave Survey (TCS, http://www.subworks.com/tcs/) and we report only the TCS cave inventory number with the cave name. General cave locations are indicated on Fig. 1 in relation to major and minor watersheds, and summaries of the cave sites are in Table 1.

**Table 1.** Detailed site descriptions of Tennessee caves sampled in this study.

Cave name	TCS no.	County	Visitation dates	Personnel	Lithology	Cave description	Water depth	Benthic habitat	Watershed
Cave Creek Cave	TRN5	Roane	28 June 2014; 3 May 2018, 3 June 2018, 3 July 2018, 15 December 2018	1^st^ trip: MLN, ASE, ETC, CDR Stephen, S Engel, A Paterson, and J Carter; Later trips: NSG and EBP	Ordovician Mascot Dolomite	Ca. 135 m traversable passage with stream, discharging as a spring entrance	5 cm to 3 m at normal flow	Primarily silt and sand with interspersed gravel and cobbles	The cave stream flows into Cave Creek, which empties directly into Watts Bar Lake on the Tennessee River.
Pedigo Cave	TKN103	Knox	14 July 2018; 27 July 2018; 15 December 2018	1^st^ & 3^rd^ trip: NSG and EBP; 2^nd^ trip: MLN and NSG	Cambrian Maynardville Limestone	Ca. 35 m of traversable passage, with stream flow in small room near cave terminus	2-meter deep pool	Fine silt, sand, and gravel mixed with larger cobble and smooth-faced rocks	The cave is within the Melton Hill Lake watershed of the Clinch River, which flows into Watts Bar Lake and the Tennessee River.
Eblen Cave	TRN6	Roane	24 March 2019	ETC, NSG, and EBP	Copper Ridge Dolomite	1,020 m of traversable passage, with 200 m of cave stream	<0.3 m deep	Larger rocks at first ~25 m, with cobble/fine silt/ sand throughout passage	The cave stream flows into Mill Creek on the surface, which is in the Clinch River watershed of the Tennessee River.

### Morphological analyses

Snails to be dissected for examination of the internal anatomy were collected, relaxed using dissolved menthol in the field, then preserved in 70% ethanol. Shells were partially dissolved in 1:1 water to hydrochloric acid, with remaining shell removed by hand. Following shell removal, tissues were immersed in Bouin’s solution as a staining fluid to enhance contrast of the tissues.

Shells and other hard parts were prepared, examined, and imaged at ANSP by NFS. Measurements in Table 2 were taken from the images of the specimens and cross checked using a calibrated ocular micrometer in a Zeiss Stemi 2000-C stereomicroscope. Following the dissection of individual holotype and paratype specimens, the shells, radulae, and opercula were prepared for mounting on SEM stubs by immersion in full strength bleach, followed by immersion in distilled water and finally 80% ethanol. Specimens were then mounted on SEM stubs, allowed to dry for 2 days, and sputter coated with gold using a Denton Desk II.

**Table 2.** Shell measurements of *A. tennesseensis* sp. nov. and Antrorbis cf. tennesseensis.

Specimen	Diameter	Height	Minimum Diameter	Whorls
ANSP 476793 (holotype)	1.75	0.77	1.40	3.15
ANSP A477042 (paratopotype)	1.35	0.58	1.12	3.2
ANSP A477042 (paratopotype)	1.17	0.63	0.93	2.75
ANSP A476794 (Pedigo Cave)	1.00	x	0.80	2.85
ANSP A476794 (Pedigo Cave)	1.18	x	0.97	3.2
ANSP A476794 (Pedigo Cave)	1.27	x	1.01	3.25

**Figure 2.**
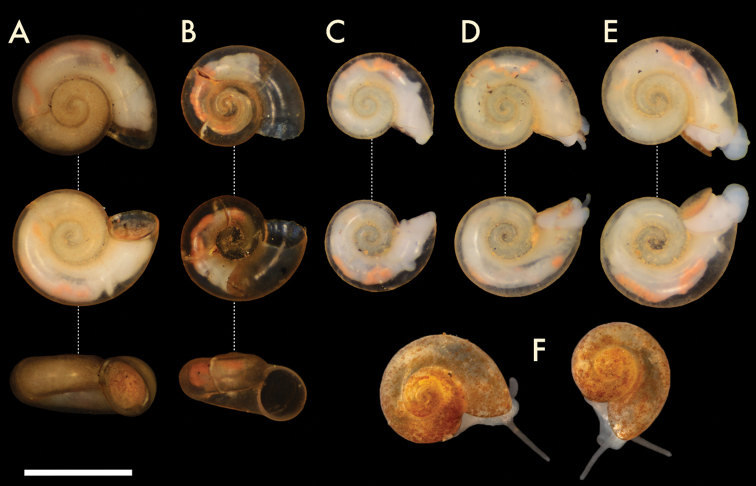
Paratype specimens of *Antrorbis tennesseensis* sp. nov. **A, B** Paratypes ANSP A477042 **C–E**Antrorbis cf. tennesseensis; ANSP A476794 **F** live specimens of Antrorbis cf. tennesseensisfrom Pedigo Cave. Scale bar: 1 mm. Photograph credits: **A–E** NFS; F MLN.

**Figure 3.**
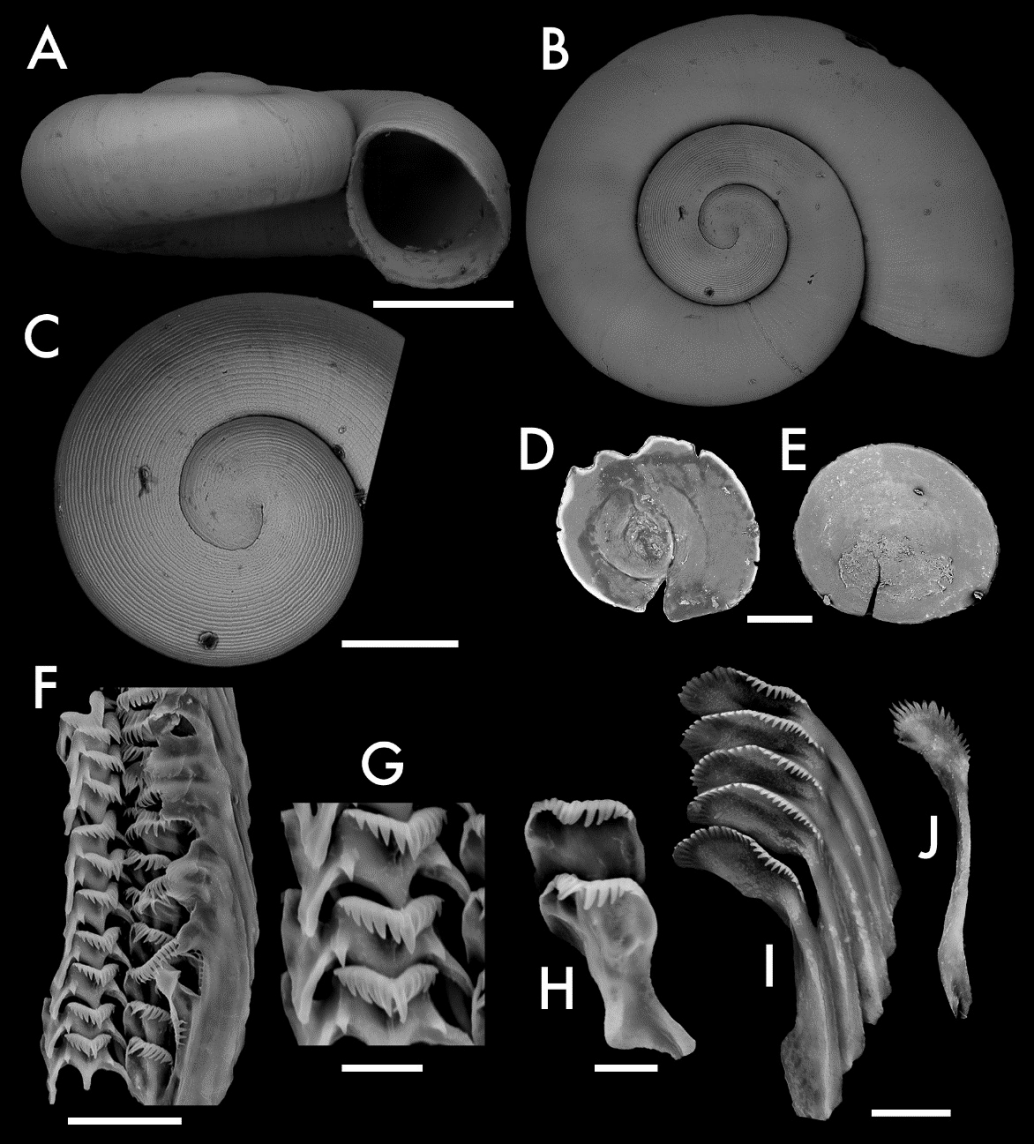
Shell, opercula, and radula of *Antrorbis tennesseensis* sp. nov. **A, B** Holotype, ANSP 476793 **C** protoconch of Antrorbis cf. tennesseensis, ANSP 476793 **D** operculum, outer side, ANSP A477042 **E** operculum, inner side, ANSP 476793 **F** radula of ANSP 476793 **G** central teeth, ANSP 476793 **H** lateral teeth, ANSP 476793 **I** inner marginal teeth, ANSP 476793 **J** outer marginal teeth, ANSP 476793 Scale bars: 0.5 mm (**A, B**); 200 μm (**C**); 200 μm (**D, E**); 20 μm (**F**); 5 μm (**G**); 5 μm (**H**); 10 μm (**I, J**). Photograph credits: NFS.

Specimen photographs for Figs 2A–E, 3 were taken by NFS using a Nikon DSLR at ANSP. The camera was controlled using HeliconRemote to take focus stacks of 3–15 images which were then combined in HeliconFocus. Brightness, color, contrast, and highlights were adjusted using the “Auto Levels” function in Lightroom Classic CC 2018 before cropping in Photoshop CC 2018. Scanning electron micrographs of shells, radulae, and opercula were taken using the Phenom G2 Pro desktop scanning electron microscope (SEM) at the Academy of Natural Sciences of Philadelphia, Drexel University. Photo- and micrographs were cropped and arranged into plates by NFS using Adobe Photoshop CC 2018.

### Molecular analyses

Genomic DNA was isolated from three specimens from two populations (Cave Creek and Pedigo) each using the Qiagen DNeasy Blood and Tissue Kit following the manufacturer’s protocol. We amplified a 658-bp fragment of the mitochondrial cytochrome oxidase subunit 1 (CO1) locus using primers LC01490 and HC02198 (Folmer et al. 1994). PCR products were purified using ExoSAP-IT (Affymetrix) and sequenced in both directions using BigDye chemistry at Eurofins MWG Operon (Louisville, KY, USA). Forward and reverse sequences were quality trimmed at the ends and assembled into contigs in DNA Baser v4.36 (Heracle BioSoft) and aligned using MUSCLE (Edgar 2004). Sequences were not generated for specimens collected from Eblen Cave due to low sample size and were thereby not included in the phylogenetic analysis. However, given geographic proximity, geological similarity, and morphological similarity, we diagnose this population as a second population of *A. tennesseensis*. CO1 sequences generated in this study were accessioned into GenBank (MN366030–MN366035). GenBank accession numbers for all other, previously published snail sequences used in this study are listed in Suppl. material 1: Table S1.

We generated a CO1 phylogeny using a maximum likelihood (ML) method in W-IQ-TREE (Trifinopoulos et al. 2016) and used its model-testing function to infer the best-fit substitution model for each codon partition under the corrected Akaike’s Information Criterion (AICc). We implemented a general time-reversible model with corrections for a discrete gamma distribution (GTR+Γ) for the first and second codon positions, and the same model with a proportion of invariant sites (GTR+Γ+I) for the third codon position. Branch support was assessed with 10,000 ultrafast bootstrap replicates (Hoang et al. 2017). Prior to the generation of the phylogeny, tests for nucleotide saturation were performed in the software package DAMBE 7.2.1 using the *I_ss_* index of saturation (Xia et al. 2003; Xia and Lemey 2009). These tests revealed no saturation among first and second codon positions (*I_ss_* = 0.5966, *I_ss.c_* = 0.7385) or third position (*I_ss_* = 0.6283, *I_ss.c_* = 0.6343). Lastly, due to the maximum likelihood topology showing ambiguous relationships among *Antrorbis* spp., we performed a Kishino–Hasegawa (KH) test (Kishino and Hasegawa 1989) and Shimodaira– Hasegawa (SH) test (Shimodaira and Hasegawa 1999) to assess the alternative *a priori* phylogenetic hypothesis that the Cave Creek Cave and Pedigo Cave are monophyletic within *Antrorbis*. These tests were implemented in PAUP* (Swofford 2002). Alternative topology was generated as a customized Newick tree file.

### Conservation assessment

We conducted IUCN Red List and NatureServe conservation assessments for *Antrorbis tennesseensis* (IUCN 2001; Master et al. 2009). We additionally include the Pedigo Cave population in these assessments, despite the ambiguous affinity of this population. Both assessments rank species into one of seven unique categories on a continuum of increasing extinction risk. Risk categories were calculated using the RAMAS Red List 3.0 (Akcakaya et al. 2007) and the NatureServe Rank Calculator v3.186 (Faber-Langendoen et al. 2012) for the IUCN Red List and NatureServe assessments, respectively. Geographic range size was calculated using two different measures for the extent of occurrence (EOO) and area of occupancy (AOO). Abundance data from all surveys were incorporated into each assessment. Additionally, we utilized the threat classification scheme proposed by Salafsky et al. (2008) to calculate an overall threat impact to this species.

## Systematic account

### Antrorbis tennesseensis

Taxon classificationAnimaliaLittorinimorphaCochliopidae

Perez, Shoobs, Gladstone, & Niemiller sp. nov.

A6A6FB0A-D6F1-52B8-9E24-F4987020ED70

http://zoobank.org/8DBE2C9C-7378-48B1-AAF8-7AD7095DEE43

Figs 1A–F , 2A–J

#### Holotype.

ANSP 476793 (one dry shell in vial, radula, and operculum on SEM stub), stream in Cave Creek Cave (TCS no. TRN5), Roane County, Tennessee, U.S.A.

#### Paratypes.

ANSP A477042 (same lot as holotype, two whole wet specimens, two dissected wet specimens, one operculum on SEM stub). Three specimens (one dry shell on SEM stub, two whole wet specimens) from Eblen Cave (TCS no. TRN6), Roane County, Tennessee, U.S.A. Specimens are currently housed at the University of Alabama in Huntsville.

#### Other examined material.

ANSP A476794 (three whole wet specimens), stream in Pedigo Cave (TCS no. TKN103), Knox County, Tennessee, U.S.A. Tentatively classified as Antrorbis cf. tennesseensis.

#### Morphological diagnosis.

A minute, planispiral *Antrorbis*, which can be readily distinguished from its sole known congener by the absence of raised tubercles on its finely spirally striate protoconch and its unique radular formula.

#### Molecular diagnosis.

Average uncorrected pairwise genetic distance at the mitochondrial CO1 locus between *A. tennesseensis* and *A. breweri* is 11.7%, with 74.5 ± 2.14 mutations separating the two species. Additionally, average uncorrected pairwise genetic distance at CO1 between the holotype *A. tennesseensis* and the Pedigo Cave population (Antrorbis cf. tennesseensis) is 9.8%.

#### Description.

Shell (Figs 2A–F; 3A–C) planispiral, 1.0–1.75 mm in diameter; 0.58–0.78 mm in height, with 2.75 to 3.25 rounded and variably descending whorls marked by deeply impressed sutures. Aperture nearly circular, almost as wide as high (0.55 × 0.56 mm in the holotype), with an internally thickened and slightly reflected peristome. Umbilicus wide, rapidly expanding. Color pale translucent yellow, with a thick, mottled yellow-orange periostracum. Protoconch (Fig. 3C) 1.8 whorls, finely spirally striate, with the innermost striae occasionally and variably punctuated or subtuberculate. Spiral striae continuing into the teleoconch weakly, intersecting with similarly weak axial growth lines.

Operculum (Fig. 3D, E) paucispiral with 4.5 whorls, ovate, thin, round, externally concave and sub-conical in profile, with a fragile, tapered periphery. Inner side smooth, with retractor muscle scar variably and roughly thickened but with no peg. Outer side covered with a noticeably thick periostracum.

Radula (Fig. 3F–J) as in its congener *A. breweri*, but differing slightly in the number of cusps on each tooth: central teeth with 11–13 cusps (5-6+1+5-6) and two basal cusps, laterals with 6–8 cusps on the outer side and 5–6 on the inner side, inner marginals with 26–30 cusps, outer marginals with 19–21 cusps. Data from ANSP 476793, the holotype.

Animal soft body is absent of pigment except for scattered clumps of black granules on stomach and digestive gland; intestine with orange, oval fecal pellets extends from terminal end of animal through body whorl. Digestive system anatomy similar to that described for *A. breweri*, including intestinal coil in anterior pallial roof exhibiting “reversed-S-shape.” Digestive gland extends for ~1 whorl. Penis simple, strap-like, tapers to a blunt distal end, not as sharply tapered as in *A. breweri*. Neither terminal papillae nor specialized penial glands observed. Testis one mass with no lobes. Seminal vesicle short and uncoiled, attaching at end of testis. Ovary an orangish mass, filling ~less than 25% of one whorl, capsule gland and albumen gland approximately equal in size, both underly and posterior to the intestine, bursa copulatrix pear shaped. Shells of individuals from Cave Creek Cave are somewhat thicker than those from Pedigo Cave and the fecal pellets are larger and more ovate in shape.

#### Etymology.

The specific epithet *tennesseensis* is in reference to this species being from the state of Tennessee. It is also a reference to the University of Tennessee in Knoxville, where several of the authors received degrees (MLN, NSG, and ETC) or are faculty (ASE). Suggested common name is Tennessee Cavesnail.

#### Distribution.

*Antrorbis tennesseensis* is known only from two caves developed within karst valleys near the confluence of the Clinch and Tennessee rivers and in upper Cambrian to lower Ordovician carbonate rocks of the Knox Group of the AVR of eastern Tennessee (Fig. 1). Cave Creek Cave and Eblen Cave are only 6 km apart from each other on opposite sides of a surface watershed divide, but cave passages could be hydrologically connected. In contrast, Pedigo Cave (Antrorbis cf. tennesseensis) is located to the northeast in northern Knox County. There could be additional caves with *A. tennesseensis* within the immediate karst area, but the potential for a significantly wider distribution for *A. tennesseensis* throughout eastern Tennessee is low due to the generally restrictive nature of folded and faulted nature of the karstified strata.

#### Ecology.

Snails are largely found amongst cobble in shallow cave streams in the dark zone. At Cave Creek Cave, several snails could be observed on a single cobble, and *Caecidotea* spp. and *Crangonyx* spp. amphipods were found on the same rocks as snails. From Pedigo Cave (Antrorbis cf. tennesseensis), a single small crayfish (*Cambarus bartonii*) was observed, but no other aquatic species were seen in the cave stream. Several snails were also found in close proximity on the same rock, with up to seven individuals on a single rock. No other fauna was found in Eblen Cave, and no egg masses or other aspects of the reproductive biology of *A. tennesseensis* have been observed from any of the caves. Although annual physicochemical measurements were not acquired in this study, the parameters measured once from each system (Table 3) were similar and within the expected conditions for water flowing through carbonate rocks.

**Table 3.** Examples of physicochemical measurements from each cave.

Cave	Sampling date	Temperature (°C)	pH	Specific conductance (µS/cm)
Cave Creek Cave	3 February 2019	14.5	6.95	253.6
Eblen Cave	24 March 2019	12.0	7.45	285.1
Pedigo Cave	27 January 2019	14.1	6.74	282.4

#### Habitat.

**Figure 4.**
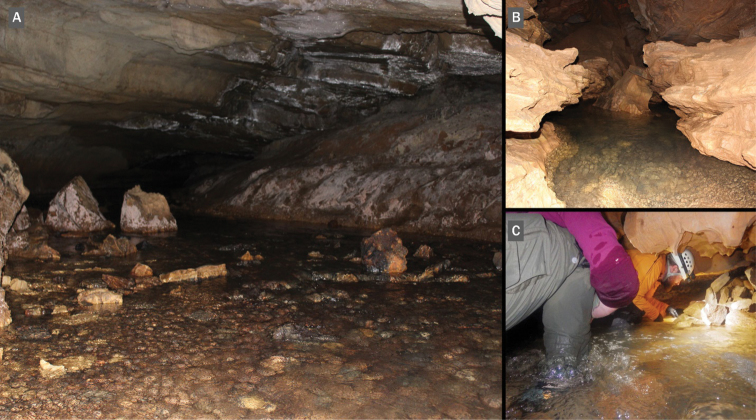
**A** Cave Creek Cave. Bottom **B** Pedigo Cave **C** Eblen Cave (NSG and ETC). Photograph credits: **A, B** NSG **C** EBP.

*Antrorbis tennesseensis* has been observed on the sides and undersurface of larger cobble and flat rocks in shallow water (< 12 cm water depth) of the stream in Cave Creek Cave and more than one meter of water depth in Pedigo Cave (Antrorbis cf. tennesseensis). At Cave Creek Cave, *A. tennesseensis* has been found only from a small (55 m^2^) area ca. 40 m from the entrance (Fig. 4A). Snails had higher abundances among small cobble substrate and larger rocks in the cave stream near the stream bank (i.e., within 0.6 m), with an average flow rate of 0.34 m/s and depth below 8 cm. At Pedigo Cave, Antrorbis cf. tennesseensis were found throughout the cave stream, and specifically in a riffle with several flat rocks and cobbles ca. 30 m from the entrance. They were also found in deeper pools under larger rocks (Fig. 4B). At Eblen Cave, only three snails were found within the primary stream passage accessible in the right fork ca. 100 m from the cave entrance. All snails were found on larger rocks semi-submerged in silt-bottom substrate, which was the primary substrate at the beginning of the stream.

#### Phylogenetic relationships.

**Figure 5.**
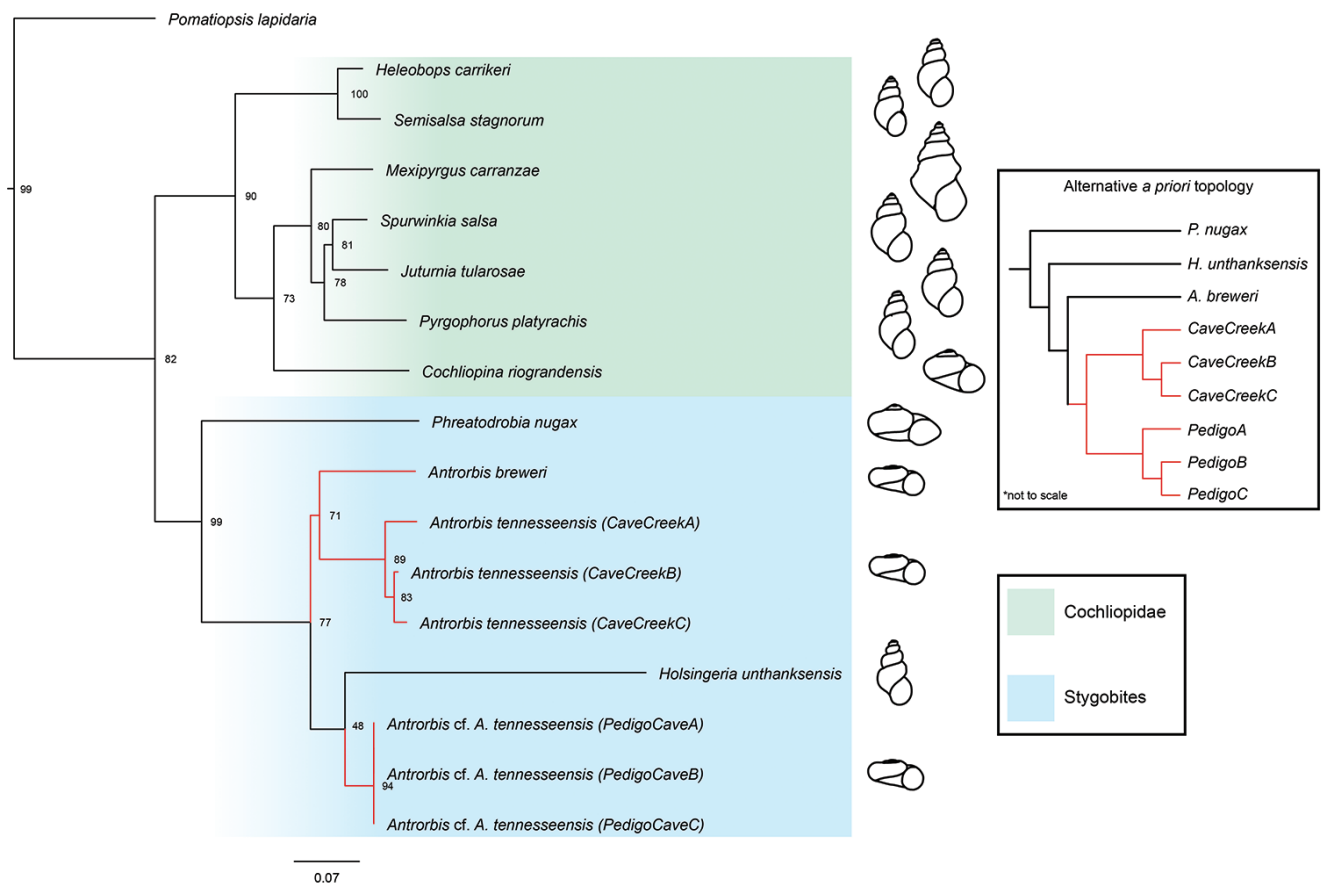
Maximum-likelihood phylogeny of four families within the Cochliopidae. Phylogeny is inferred by W-IQ-TREE based on mitochondrial CO1 sequences (658 bp aligned). Ultrafast bootstrap support values shown at nodes.

The resulting CO1 gene topology of the ML phylogeny is shown in Fig. 5. The genus *Antrorbis* clusters with the stygobitic Cochliopidae along with the recently reassigned *Phreatodrobia* (Clark 2019a) and the genus *Holsingeria*. The holotype population of *A. tennesseensis* forms a clade with *A. breweri*. population, but forms a paraphyletic group with the morphologically similar Pedigo Cave population (Antrorbis cf. tennesseensis) in the maximum likelihood phylogeny. However, results of the KH and SH tests fail to reject the *a priori* topology of monophyly (*P* > 0.05 for both tests).

#### Conservation assessment.

This species is currently only known from two caves that are within geologically and hydrologically restricted strata. However, the third cave population for Antrorbis cf. tennesseensis was incorporated in these analyses. The NatureServe rank calculated for *Antrorbis tennesseensis* is Critically Imperiled (G1). For the IUCN Red List assessment, *A. tennesseensis* was assessed as Endangered (EN) B1a, as the species is known from fewer than five sites and has an EOO <5,000 km^2^. The AOO was calculated as 12.0 km^2^ and EOO was 73.3 km^2^. A maximum of 23 (on 3 June 2018), 54 (on 15 December 2018), and 3 individuals (on 24 March 2019) were observed at Cave Creek, Pedigo, and Eblen Caves, respectively. Mean number of snails observed at Cave Creek Cave and Pedigo Cave (for which multiple surveys were conducted) was 18.4 ± 4.3 and 36 ± 7.8, respectively. Overall, threat impact for this species was calculated as Very High, with the two most outstanding categories of the threat assessment being Human Intrusion and Disturbance and Pollution.

## Discussion

We describe a new species of stygobitic snail, *Antrorbis tennesseensis*, which is distinguished from its only congener *A. breweri* by the absence of raised tubercles on the spiral striae on the external protoconch and substantial divergence at the mitochondrial CO1 locus (holotype, Cave Creek Cave). Given the geographic proximity, geological similarity, and identical morphology, we additionally diagnose the Eblen Cave population as a second population of *A. tennesseensis*. *Antrorbis tennesseensis* and *A. breweri* form a modestly supported clade in the ML phylogeny. Individuals from the third population discovered in this region (Pedigo Cave), despite being morphologically similar to *A. tennesseensis*, form a paraphyletic group with *A. tennesseensis* in the ML phylogeny. In the ML phylogeny, the Pedigo Cave population shows closer genetic similarity to *Holsingeria unthanksensis*, another stygobitic snail in the northern AVR that was initially thought to form a clade with *Antrorbis* due to morphological similarity (Hershler and Holsinger 1990). However, this relationship is not a strong one and given the failure to reject alternative topologies by both the KH and SH tests, we tentatively classify this third population as Antrorbis cf. tennesseensis and encourage subsequent molecular study to understand the identity of this population and the systematics of the stygobitic group. A plausible explanation may be that *Antrorbis* and *Holsingeria* are more closely related than previously understood, despite their disparate morphologies.

Within the superfamily Truncatelloidea, observing paraphyly and high intraspecific divergence among species within other gastropod families (especially subterranean groups) is common when analyzing mitochondrial genes (Wilke et al. 2013; Whelan and Strong 2016; Gladstone et al. 2019). As a result, we cautiously provide these molecular results as only one of several lines of evidence for the species designation of *A. tennesseensis* and advise for the incorporation of additional genetic data in the future. Currently, our robust morphological diagnoses provide ample distinctions between *A. breweri* and *A. tennesseensis*, in addition to their contrasting geography and ecology.

These two *Antrorbis* species are separated by ca. 250 km and occur in caves developed in different strata and in different major hydrological river basins, as *A. tennesseensis* is distributed within the Tennessee River watershed, which flows into the Ohio River, and *A. breweri* is from Manitou Cave in the Coosa River watershed that flows directly into the Gulf of Mexico. However, from the Eocene (55 million years ago) through the mid-Miocene, the ancestral Appalachian River occupied the drainage basins of the Tennessee and Coosa river and flowed through the southern AVR, across Alabama, and emptied into Mobile Bay at the Gulf of Mexico (Milici 1968; Galloway et al. 2011; Hoagstrom et al. 2013). Cave sediment records in Tennessee (Anthony and Granger 2007) and distinct changes in Gulf of Mexico delta sedimentation histories for the Mississippi and Coosa rivers in the mid-Miocene to late Pliocene point to major redistribution of the watersheds (Galloway et al. 2011). The modern drainage divide between the Tennessee and Coosa rivers occurs in northern Alabama, near the location of Manitou Cave. Although the timing of Tennessee-Coosa river divide development is not well understood, a number of processes likely contributed to the separation of the two rivers, including uplift of the southern Appalachian Mountains and subsequent incision of local streams (Gallen et al. 2013), stream downcutting to accommodate rapid base-level drops due to widespread glaciation in the northern hemisphere, and stream piracy through Walden Ridge on the edge of the Cumberland Plateau and into the karstified Sequatchie Valley in Tennessee (Johnson 1905; Clark 1989; Self 2000).

Based on the modern distribution of *Antrorbis*, the common ancestor for the two currently known species must pre-date the late Pliocene emergence of the Tennessee-Coosa drainage divide. Similar timing for the isolation of distinct genetic lineages of the Southern Cavefish, *Typhlichthys subterraneus*, in the Tennessee and Coosa river drainages has also been proposed (Niemiller et al. 2016a). Considering the widespread modern distribution of the genus, and possible paleogeographic explanation for its distribution, it is possible that other *Antrorbis* species currently exist in caves within the modern Tennessee-Coosa river basins. It is also possible that the distinct modern cave populations of *A. breweri* in Alabama and *A. tennesseensis* in Tennessee are relict populations of a much more regionally widespread genus. In the future, evidence of their distribution and for the timing for Tennessee-Coosa river divide development could come from paleontological investigations of cave sediments with dateable material (e.g., organic matter, shell material), which could include the snails themselves.

### Implications for classification

Original designation of the stygobitic genera *Antrorbis*, *Holsingeria*, and *Phreatodrobia* to Lithoglyphidae was based on soft tissue anatomy (Hershler and Thompson 1990), which was also supported by their similar morphology and subterranean ecology (Hershler and Holsinger 1990). The addition of molecular data has drastically revised our understanding of relationships among the hydrobioid lineages. The most recent molecular treatment (Wilke et al. 2013) supports the division of the former Hydrobiidae into several smaller families. The results of our phylogeny show high support for this stygobitic clade among other members of the Cochliopidae from the Wilke et al. (2013) analysis. As such, we tentatively place the genus *Antrorbis* in the family Cochliopidae.

### Conservation implications

Freshwater snails have experienced significant declines globally, with nearly 93% of all recorded extinctions being narrow endemic species (Lydeard et al. 2004; Johnson et al. 2013). Many stygobitic taxa exhibit high levels of endemism, with notable sensitivity to environmental alteration (Culver and Pipan 2009; Niemiller and Zigler 2013). There are many unique threats to subterranean environments and their associated taxa, with the conversion of the surface landcover for urban development and agriculture being most prominent (Culver and Pipan 2009). Populations of *A. tennesseensis* are found near roadways and suburban neighborhoods, and, therefore, may be at an increased risk of extirpation due to sedimentation, changes in local hydrology, and chemical effluent runoff into their cave streams. Further, despite occurring on private lands, these caves experience frequent visitation from recreational cavers. Vehicles can often be seen in proximity to Cave Creek Cave, and an array of plastics, scrap metals, and other waste materials can be found in each of the cave systems. Future consideration should be given for more deliberate protection and conservation of *A. tennesseensis*.

## Supplementary Material

XML Treatment for Antrorbis tennesseensis

## Supplementary materials

Supplementary material 1 Table S1. GenBank accession numbers for all taxa utilized for the phylogenetic analysis Data type: species data https://binary.pensoft.net/file/363808This dataset is made available under the Open Database License (http://opendatacommons.org/licenses/odbl/1.0/). The Open Database License (ODbL) is a license agreement intended to allow users to freely share, modify, and use this Dataset while maintaining this same freedom for others, provided that the original source and author(s) are credited.Nicholas S. Gladstone, Kathryn E. Perez, Evelyn B. Pieper, Evin T. Carter, Katherine E. Dooley, Nathaniel F. Shoobs, Annette S. Engel, Matthew L. Niemiller
